# Impact of Nuun Electrolyte Tablets on Fluid Balance in Active Men and Women

**DOI:** 10.3390/nu12103030

**Published:** 2020-10-02

**Authors:** Jacquelyn Pence, Richard J. Bloomer

**Affiliations:** 1 Center for Nutraceutical and Dietary Supplement Research, University of Memphis, 161 Roane Fieldhouse, Memphis, TN 38152, USA; jpence1@memphis.edu; 2 College of Health Sciences, University of Memphis, 106 Roane Fieldhouse, Memphis, TN 38152, USA

**Keywords:** hydration, electrolytes, sport drink, athletes, fluid balance

## Abstract

Background: Maintaining adequate hydration is important for overall health and has major implications for athletes involved in physically demanding tasks. While water is viewed as an effective means to rehydrate, and is inexpensive and readily available, electrolyte beverages appear to be more beneficial, in particular for athletes who routinely lose electrolytes through sweating. Nuun tablets contain a mix of electrolytes and are quickly dissolved in water to create an electrolyte-rich beverage. We determined the impact of Nuun tablets on the fluid balance of healthy, exercise-trained men and women at rest. Methods: Eight men (25.9 ± 4.5 yrs) and 10 women (28.2 ± 9.4 yrs) ingested either water only or water with Nuun electrolyte tablets, at both a single and double strength concentration, in random order, on three occasions separated by approximately one week, in a fasted and euhydrated state. A total of 1 L of fluid was ingested at each visit over a 30 min period. Urine was collected from each subject at 0, 1, 2, 3, and 4 h post-ingestion. Urine mass values were used to calculate fluid balance and the beverage hydration index (BHI; i.e., the volume of urine produced after drinking the Nuun beverages, relative to that of water only—control condition). Heart rate and blood pressure were measured throughout the four-hour period, while body weight was measured at the start and end of the experiment. Results: Neither heart rate nor blood pressure were impacted by beverage consumption. Nuun tablets resulted in a lower urine output compared to water, with fluid balances for both concentrations more favorable compared to water (*p* < 0.05), beginning at 2 h post-ingestion and continuing at the 3 h and 4 h times. Body weight loss was less with Nuun at the single dose (0.38 kg; *p* = 0.02) and double dose (0.43 kg; *p* = 0.08), compared to water (0.57 kg). The BHI was higher for Nuun (single dose in particular) compared to water at both 2 h (*p* = 0.05) and 4 h (*p* = 0.02). Conclusion: The addition of Nuun electrolyte tablets to water improves the fluid balance and BHI in healthy men and women. Results were similar for both concentrations, suggesting that additional electrolytes are not necessary when in a rested state. Future studies should determine the impact of various concentrations of the Nuun beverage during physical exercise—in particular, exercise in the heat, when sweat loss may be highest.

## 1. Introduction

Maintaining adequate hydration is essential to optimal health [1,2,3] and athletic performance [4,5]. Dehydration can contribute to a host of issues, including altered cognition and digestion, as well as organ dysfunction in the heart, kidneys, and skin [3]. When individuals exercise (particularly in a warm environment), they can lose excessive amounts of fluids through sweating, along with necessary electrolytes (e.g., sodium, potassium, chloride) [5,6]. With dehydration, athletes may feel sluggish and physical performance can suffer [7,8].

Many attempts have been made to improve the hydration status of active individuals [7,8,9,10]. According to the American College of Sports Medicine’s position stand, fluids containing sodium should be slowly ingested leading up to activity to help retain fluids, so that athletes maintain euhydration with normal electrolyte levels [6]. Hydration strategies during physical activity should be aimed at preventing fluid loss greater than 2% of the body weight, which can be optimally accomplished by ingesting a diluted carbohydrate/electrolyte beverage at a rate approximately equivalent to sweat loss. During recovery, enough water and food containing sodium should be ingested to replenish electrolyte losses and reestablish euhydration [6]. This approach seems to work well. However, some debate remains over what the best fluid is to consume, particularly with respect to macronutrient type and the specific electrolyte mix.

Related to the above, it is well-accepted that electrolyte replenishment is of importance both during and following exercise, to aid in rehydration for subsequent exercise bouts [6,11]. Electrolytes (sodium in particular) have been used for decades to aid athlete hydration, and this has led to the development of various sport drinks—which also often include moderate amounts of carbohydrate (e.g., Gatorade^®^ (PepsiCo; Chicago, IL, USA) and Powerade^®^ (The Coca-cola Company; Austin, TX, USA)). However, one problem with carbohydrate ingestion is that some individuals experience gastrointestinal (GI) upset following carbohydrate ingestion before [12] and during an event [13], despite very good physical performance outcomes. Due to this GI upset, some individuals (in particular, recreationally active individuals who are not competing at high levels) rely solely on water and seek a method to ingest the lost electrolytes. In addition, some individuals prefer to have both plain water and an electrolyte beverage during their training/competition sessions, and in some activities (e.g., running and cycling), carrying multiple bottles of fluid is difficult.

The solution to the above issues for many athletes is the use of electrolyte tablets. These can be dropped into plain water and will dissolve into a flavored electrolyte-rich beverage. They are easy to transport and provide the needed electrolytes to replace those that are lost through intense and/or long duration exercise.

Nuun electrolyte tablets contain 2 g of carbohydrates and a combination of electrolytes, provided at a relatively low percentage of the daily value (DV). While these tablets are commercially available and have received positive reviews from end users, there have been no studies to date to evaluate the impact of these tablets on fluid balance. Therefore, the purpose of this study was to determine the impact of Nuun electrolyte tablets on fluid balance in young and active men and women. We followed a similar approach as used by Maughan and colleagues to measure the fluid balance and the beverage hydration index (BHI) [14]. It was hypothesized that Nuun electrolyte tablets would result in a more favorable fluid balance compared to water only. Due to the fact that athletes use Nuun tablets at varying dosages, we also evaluated Nuun at both a single concentration and double concentration. We maintained a non-directional hypothesis regarding the potential differences between the two different dosages tested.

## 2. Materials and Methods

A total of 8 men and 10 women were enrolled and completed this crossover study. All procedures were approved by the University of Memphis Institutional Review Board for Human Subjects Research (protocol FY2020-327), and the study was registered through ClinicalTrials.gov (NCT04422158). Subjects were required to be between 18 and 45 years old, non-tobacco users, not obese (body mass index under 30 kg/m^2^), willing to refrain from alcohol and caffeine within 48 h of each test day, willing to refrain from strenuous exercise for 24 h prior to each test day, without active infection of any kind, consuming two or more liters of fluid daily, engaged in structured exercise three or more hours per week, and if female, not pregnant. Subjects were paid $150 for their full participation.

### 2.1. Screening Visit

During the initial visit to the laboratory, subjects completed the informed consent form, health history, medication and dietary supplement usage, and physical activity questionnaires. Subjects’ heart rate and blood pressure, height, weight, waist, and hip circumference were measured. To confirm non-pregnancy, females were provided with a urine pregnancy test kit (Clinical Guard^®^; Atlanta, Georgia, USA), escorted to a private restroom (within the lab), and asked to perform the test. Eligible subjects were scheduled for weekly testing visits after screening was completed. 

### 2.2. Independent Variable

Nuun electrolyte tablets were evaluated in this investigation. These tablets provide carbohydrate and a combination of electrolytes at a relatively low percentage of the daily value (DV). Specifically, Nuun tablets each contain roughly 15 calories and 2 g of carbohydrate, in addition to calcium (13 mg, 1% DV), sodium (300 mg, 13% DV), potassium (150 mg, 3% DV), magnesium (25 mg, 6% DV), and chloride (40 mg, 2% DV), thus providing adequate electrolytes to replace losses as a result of physical activity.

Both a single and double concentration of the Nuun tablets were provided to subjects, on two separate days, approximately one week apart. The tablets were dissolved in cold bottled water. Specifically, and on request from the study sponsor, for the single concentration, three tablets were dissolved in 1.4 L of water, and the subjects consumed 1 L of the solution. For the double concentration, six tablets were dissolved in 1.4 L of water, and the subjects consumed 1 L of the solution. Since Nuun packaging recommends that one tablet be dissolved into 500 mL of water, we essentially provided double the recommended fluid volume. A cold bottled water (control) condition was also included in the design and ingested at 1 L. For all three conditions, subjects ingested the fluid over the course of a 30 min period (250 mL per 7.5 min). Therefore, a total of three different conditions were included as follows, in random order and using a single-blind design: water only; water + Nuun (single concentration); water + Nuun (double concentration). All subjects received each condition, using a crossover design, with approximately one week separating conditions. The same brand of bottled water was used for all conditions. Subjects reported to the lab following an overnight fast (>10 h) and in a euhydrated state. They were instructed to void (empty bladder) upon waking. They were provided with a 16 ounce bottle of water to consume one hour prior to their scheduled lab visit time. They reported to the lab in the morning hours (e.g., 6:00–7:30 a.m.) and rested quietly upon arrival for 10 min. They were instructed to void their bowels and bladder, as needed. Body weight was then measured, wearing only underwear and a standard medical gown. Subjects were then provided with the assigned beverage as noted above. There was no attempt made to alter the color or taste of the water. One fourth of the beverage was provided every 7.5 min for ingestion (1 L total volume) over a 30 min period. Participants were asked to empty their bladder at the end of the 30 min drinking period (0 h) and again at the end of each hour of the study period after the 0 h time (total of 4 h). Therefore, the total time of subject participation from the start of beverage consumption to the collection of the 4 h urine collection was 4.5 h. If a participant requested to pass urine before the hour was complete, this was collected and then added to any further urine produced at the end of the corresponding hour.

In addition to the collection of urine at the end of each hour, the heart rate and blood pressure of subjects was measured and recorded. After the final urine sample was collected, body mass was recorded again (wearing only underwear and gown). No additional food or calorie containing beverages were allowed throughout the study period. Subjects remained in the lab during the entire time and were supervised throughout.

### 2.3. Urine Collection

All urine was collected by subjects in a private restroom and passed into a urine collection container, which was then provided to the investigators for measurement using a standard laboratory balance. This approach was similar to that used by Maughan and colleagues [14] to measure the fluid balance and BHI. While additional measures, such as changes in plasma volume, total body water, and various urine measures (e.g., specific gravity) may be of interest, these were not included in the present study.

### 2.4. Dietary Intake and Physical Activity

Subjects followed their usual activity patterns over the course of the study period, but refrained from strenuous activity for the 24 h preceding each lab test day. Dietary intake was similar over the entire study period. However, subjects consumed the same standard prepackaged meals during the day prior to each test day. These included meal replacement drinks (Orgain^®^; Irvine, CA, USA), food bars (e.g., Clif Builder^®^, Clif Bar & Company; Emeryville, CA, USA), fruit, and mixed nuts (Emerald^®^, Diamond Foods, Inc.; San Francisco, CA, USA). Each subject received an allotment of these items, based on preference (e.g., three meal replacement drinks, three food bars, three pieces of fruit, and two packs of mixed nuts). They were then given the same items for visits two and three; following the same food plan for the days prior to all three lab test days. No other food or calorie-containing beverages should have been consumed during the day prior to each lab visit, other than what was provided to subjects by the investigators. However, subjects were allowed to consume as much water as they preferred.

### 2.5. Data Analysis

The data are presented as mean ± SD. Most data were analyzed using a 3 (condition) × 5 (time) analysis of variance (ANOVA), while BHI was analyzed using a one-way ANOVA. Tukey post-hoc testing and contrast analysis was used as appropriate. Analyses were performed using JMP^®^ software (version 15.1.0, SAS Institute Inc.; Cary, NC, USA), and statistical significance was set at *p* ≤ 0.05.

## 3. Results

### 3.1. Overview

Eighteen subjects, described in Table 1, successfully completed the study. None of these subjects reported a problem with beverage consumption, and all beverages were well-tolerated. A total of 20 subjects were initially enrolled in the study. However, one subject decided to cease participation due to the dietary restrictions, while one other subject was removed from the study due to an infection. Data from these two individuals are not included in the analysis. As the response was similar for both men and women, coupled with the fact that we had unequal numbers between genders, data were analyzed collectively and presented as such.

### 3.2. Heart Rate and Blood Pressure

**Figure 1:**
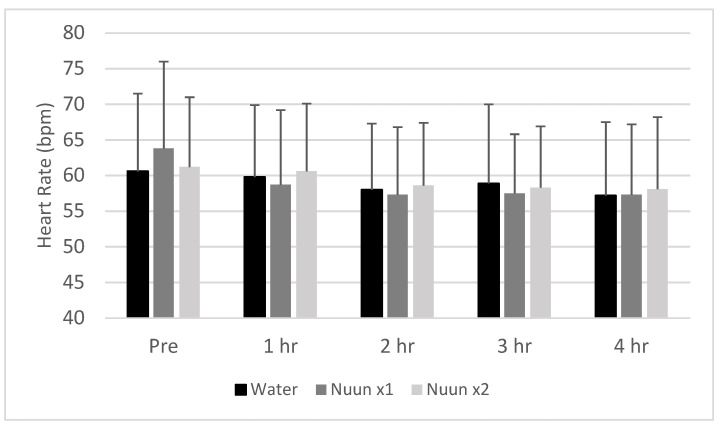
Heart rate response of 18 men and women to water only and Nuun+water; Values are mean ± standard deviation; bpm is beats per min, hr is hour(s) after condition ingestion, Nuun x1 is Nuun beverage at normal concentration, Nuun x2 is Nuun beverage at double concentration; No differences of statistical significance were noted (*p* > 0.05).

**Figure 2:**
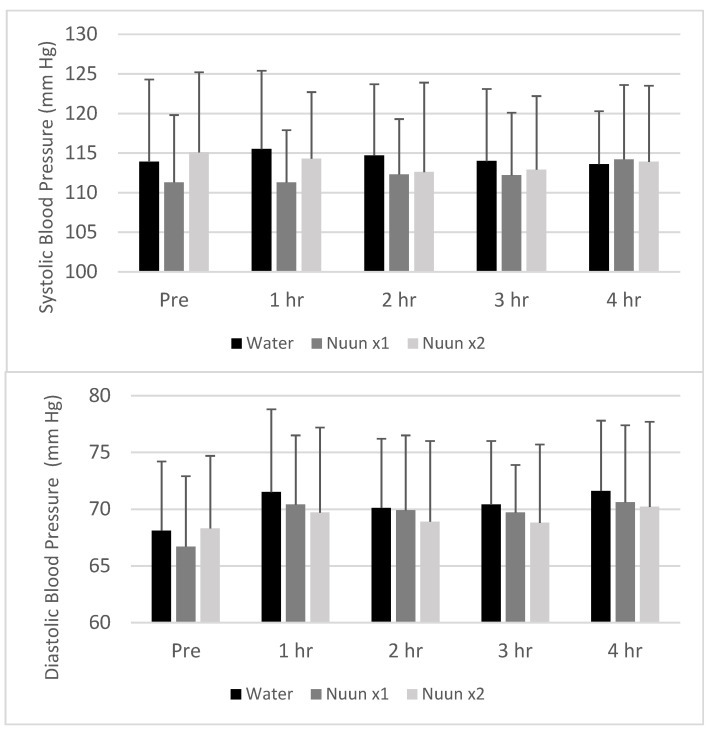
Systolic and diastolic blood pressure response of 18 men and women to water only and Nuun+water; Values are mean ± standard deviation; mm Hg is mm Mercury, hr is hour(s) after condition ingestion, Nuun x1 is Nuun beverage at normal concentration, Nuun x2 is Nuun beverage at double concentration; No differences of statistical significance were noted (*p* > 0.05).

No condition, time, or condition × time interactions were noted for heart rate or blood pressure (*p* > 0.05). Values remained relatively stable across time and were not different between conditions. Data for heart rate are presented in Figure 1, while those for blood pressure are presented in Figure 2.

### 3.3. Hydration Outcomes

**Figure 3:**
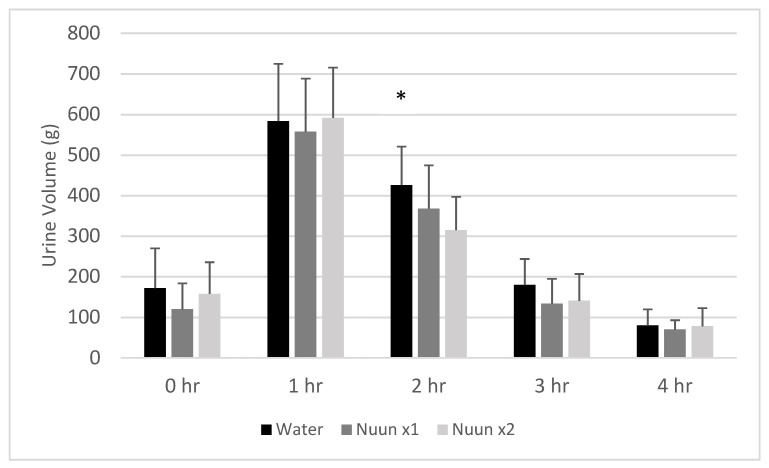
Urine volume of 18 men and women at individual times through four hours after consuming water only and Nuun+water; Values are mean ± standard deviation; hr is hour(s) after condition ingestion, Nuun x1 is Nuun beverage at normal concentration, Nuun x2 is Nuun beverage at double concentration; * Water higher than Nuun x1 (*p* = 0.05) and Nuun x2 (*p* = 0.0002); No other differences of statistical significance noted (*p* > 0.05).

A condition effect was noted for urine volume (*p* = 0.01), with values for Nuun x1 and Nuun x2 lower than water (*p* < 0.05). A time effect was also noted (*p* < 0.0001), with hours 1 and 2 higher than 0 h, and hour 4 lower than 0 h (*p* < 0.05). A trend for an interaction effect was noted (*p* = 0.14), with values for water higher than Nuun x1 (*p* = 0.05) and Nuun x2 (*p* = 0.0002) at the 2 h time. Data for urine volume are presented in Figure 3.

**Figure 4:**
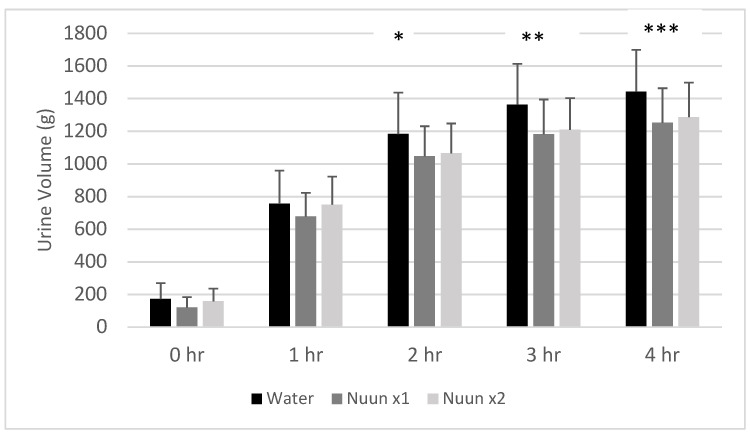
Cumulative urine volume of 18 men and women through four hours after consuming water only and Nuun+water; Values are mean ± standard deviation; hr is hour(s) after condition ingestion, Nuun x1 is Nuun beverage at normal concentration, Nuun x2 is Nuun beverage at double concentration; * Water higher than Nuun x1 (*p* = 0.03) and Nuun x2 (*p* = 0.07); ** Water higher than Nuun x1 (*p* = 0.004) and Nuun x2 (*p* = 0.02); *** Water higher than Nuun x1 (*p* = 0.003) and Nuun x2 (*p* = 0.01); No other differences of statistical significance noted (*p* > 0.05).

A condition effect was noted for cumulative urine volume (*p* < 0.0001), with values for Nuun x1 and Nuun x2 lower than water (*p* < 0.05). A time effect was also noted (*p* < 0.0001), with hours 1, 2, 3, and 4 having results higher than 0 h (*p* < 0.05). No interaction effect was noted (*p* = 0.59); however, a priori contrasts revealed multiple differences between conditions at selected times. At hour 2, water was higher than Nuun x1 (*p* = 0.03) and Nuun x2 (*p* = 0.07). At hour 3, water was higher than Nuun x1 (*p* = 0.004) and Nuun x2 (*p* = 0.02). At hour 4, water was higher than Nuun x1 (*p* = 0.003) and Nuun x2 (*p* = 0.01). Data for cumulative urine volume are presented in Figure 4.

**Figure 5:**
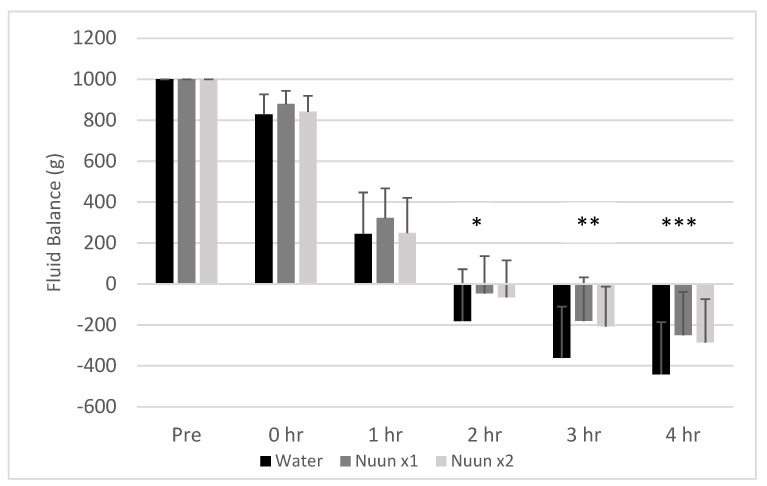
Fluid balance of 18 men and women through four hours after consuming water only and Nuun+water; Values are mean ± standard deviation; hr is hour(s) after condition ingestion, Nuun x1 is Nuun beverage at normal concentration, Nuun x2 is Nuun beverage at double concentration; * Water lower than Nuun x1 (*p* = 0.02) and Nuun x2 (*p* = 0.04); ** Water lower than Nuun x1 (*p* = 0.002) and Nuun x2 (*p* = 0.008); *** Water lower than Nuun x1 (*p* = 0.001) and Nuun x2 (*p* = 0.007); No other differences of statistical significance noted (*p* > 0.05).

A condition effect was noted for fluid balance (*p* < 0.0001), with values for Nuun x1 and Nuun x2 being higher than water (*p* < 0.05). A time effect was also noted (*p* < 0.0001), with all hours lower than baseline (*p* < 0.05). No interaction effect was noted (*p* = 0.29); however, a priori contrasts revealed multiple differences between conditions at selected times. At hour 2, water was lower than Nuun x1 (*p* = 0.02) and Nuun x2 (*p* = 0.04). At hour 3, water was lower than Nuun x1 (*p* = 0.002) and Nuun x2 (*p* = 0.008). At hour 4, water was lower than Nuun x1 (*p* = 0.001) and Nuun x2 (*p* = 0.007). Data for fluid balance are presented in Figure 5.

Based on the above responses, an effect was noted for body weight loss (*p* = 0.05), which was greater with the water condition (0.57 kg) as compared to Nuun x1 (0.38 kg; *p* = 0.02) and Nuun x2 (0.43 kg; *p* = 0.08).

**Figure 6:**
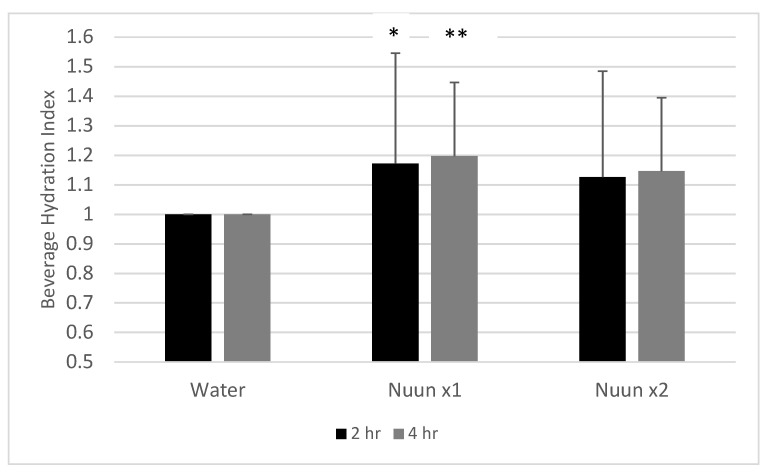
Beverage Hydration Index of 18 men and women at two hours and four hours after consuming water only and Nuun+water; Values are mean ± standard deviation; hr is hour(s) after condition ingestion, Nuun x1 is Nuun beverage at normal concentration, Nuun x2 is Nuun beverage at double concentration; * Water lower than Nuun x1 at 2 h (*p* = 0.05); ** Water lower than Nuun x1 at 4 h (*p* = 0.02); Trend for water lower than Nuun x2 at 2 h (*p* = 0.15) and 4 h (*p* = 0.09); No other differences of statistical significance noted (*p* > 0.05).

Finally, the BHI at the 2 h mark was not of statistical significance (*p* = 0.13), but contrasts revealed a higher value for Nuun x1 as compared to water (*p* = 0.05). At the 4 h mark, values approached statistical significance (*p* = 0.06), with values for Nuun x1 (*p* = 0.02) and Nuun x2 (*p* = 0.09) higher than water. Data for hydration index are presented in Figure 6.

## 4. Discussion

Findings from this study indicate that the consumption of Nuun electrolyte tablets with water can lead to improved fluid balance over the consumption of water alone in active individuals. This may be attributed to the electrolytes present within the Nuun solutions [5,14,15]. The 2 h BHI for 1× and 2× concentration Nuun electrolyte in water is similar to that previously reported for cola, and greater than that reported for items such as diet cola, sports drink, lager, coffee, and tea [14]. Carbohydrates can promote hydration by encouraging consumption, as well as delaying or decreasing diuresis [5,14]. However, as carbohydrates can lead to gastrointestinal upset [12,13], it is possible that some athletes may benefit from consuming Nuun electrolyte tablets with water over beverages with higher carbohydrate loads, such as orange juice and milk.

Interestingly, in the work of Maughan and colleagues, water was noted to be similar to the tested sports drink [14]. Those findings, compared to those of the present study, could be due to differences in the formulations of the two beverages, with Nuun having fewer carbohydrates and more electrolytes. Previous BHI research on differences between young and older adults found that increases in concentration of sodium, not amino acids or carbohydrates, resulted in greater hydration [15]. However, no significant differences were observed between the 1× and 2× concentrations of Nuun, suggesting that there may be a limit to what electrolyte levels are needed to improve fluid retention in a rested state.

No hemodynamic differences were observed across conditions. While dehydration has been shown to impact hemodynamics during [16] and after exercise [17], subjects in the current study were at rest throughout the experiment. Future studies involving an exercise condition are needed to better understand the heart rate and blood pressure response associated with varying degrees of hydration following the use of Nuun tablets.

Our data indicate that Nuun electrolyte tablets with water at 1× or 2× concentrations have comparable effects on hydration when at rest and better effects than that of water alone. Future work should focus on differences in hydrating effects between these conditions during physical exertion, where sweating contributes to considerable water and electrolyte losses. In such cases, electrolyte losses may result in differences in hydration capabilities between 1× and 2× concentration. Additionally, excessive consumption of water to rehydrate following prolonged sweating (such as during extended physical activity in heat) can lead to hyponatremia, while using a beverage high in sodium but lacking other electrolytes can result in an electrolyte imbalance, such as decreased potassium levels [5]. While sodium has been shown to promote hydration of the extracellular water compartment, potassium is important to the hydration of the intracellular water compartment and is therefore also important to hydration. It is therefore important to have a well-balanced electrolyte solution for rehydration. Additionally, other physiological processes that effect hydration, including renal function and fluid absorption in the small intestines are altered during exercise. This may cause differences in the hydrating abilities of the two concentrations of Nuun [18,19].

Beyond hydration, electrolytes are important to athletic performance and general health. Electrolytes are important to muscle, neural, and cardiovascular function [20,21,22]. Electrolyte imbalances may lead to muscle cramps and hyponatremia, leading to a higher risk of serious injuries, including coma, respiratory arrest, and death [6,22]. Electrolyte imbalances with dehydration can also lead to rhabdomyolysis. Using a well-balanced electrolyte solution, such as Nuun, along with water may help in maintaining an optimal electrolyte balance. Exercise in heat can also lead to hypotension, diminishing the nutrient availability to muscle and possibly leading to heat stroke. The effect of Nuun electrolyte solutions on hemodynamics with exercise in the heat should also be considered in future work.

While this study focused on people who exercise regularly, the findings may be extended to members of the general adult population who are seeking to improve their hydration. It is important to note that maintaining hydration requires continually consuming beverages and food to replenish water loss. As Figure 5 highlights, on average, the liter of fluid consumed prior to time 0 was excreted by the end of the second hour, and additional water continued to be lost at hours 3 and 4, resulting in a net water loss through urine excretion alone by the end of the study, without any physical activity. To maintain a level of hydration even at rest requires water to continually be consumed, particularly if individuals are not frequently drinking or eating water-rich foods and beverages. Consuming beverages like orange juice, skim milk, and some common sports drinks to maintain hydration would require a significant amount of the daily caloric intake, especially for less active individuals. Nuun could therefore provide a low-calorie alternative to improve overall hydration. Additionally, people who regularly do physically demanding work—especially in heat—may need to replenish lost electrolytes and could also benefit from drinking Nuun.

## 5. Conclusions

The findings from this study indicate that the addition of Nuun electrolyte tablets to water improves the fluid balance and BHI in a sample of healthy, active men and women. Results were similar for both dosages, suggesting that the additional electrolytes do not provide further benefit when individuals are in a rested state. Future studies involving physical exercise are needed to determine the impact of Nuun tablets, at varying dosages, on fluid balance in active men and women—in particular those who lose significant fluid due to physical activity and/or work performed in a hot environment.

## Figures and Tables

**Table 1:** Characteristics of healthy men and women.

Variable	Men (*n* = 8)	Women (*n* = 10)
Age (years)	25.9 ± 4.5	28.2 ± 9.4
Height (cm)	174.6 ± 7.5	165.8 ± 6.1
Weight (kg)	74.7 ± 10.7	60.7 ± 8.5
Body Mass Index (kg/m^2^)	24.4 ± 2.3	22.1 ± 3.1
Waist Circumference (cm)	80.3 ± 10.6	71.2 ± 7.4
Hip Circumference (cm)	100.4 ± 7.5	97.0 ± 6.1
Waist:Hip	0.79 ± 0.06	0.74 ± 0.07
Resting Heart Rate(bpm)	68.5 ± 1.7	66.7 ± 9.3
Resting Systolic Blood Pressure (mm Hg)	126.5 ± 14.1	112.3 ± 9.1
Resting Diastolic Blood Pressure (mm Hg)	75.8 ± 9.1	72.4 ± 7.8
Anaerobic Exercise (days/wk)	3.6 ± 1.9	2.7 ± 1.3
Anaerobic Exercise (min/session)	50.0 ± 22.6	41.6 ± 12.8
Aerobic Exercise (days/wk)	3.9 ± 1.8	4.6 ± 1.5
Aerobic Exercise (min/session)	46.9 ± 16.6	53.6 ± 18.4

Values are Mean ± Standard Deviation.
